# Analysis of molecular subtypes and prognostic signature of senescence-associated secretory phenotype in pancreatic cancer

**DOI:** 10.7717/peerj.20476

**Published:** 2026-01-06

**Authors:** Yuewen Kuang, Mingkun Jia, Yuming Zhu, Zhiyong Xiang

**Affiliations:** Hepatobiliary and Pancreatic Surgery, The Third Affiliated Hospital of Chongqing Medical University (Fangda Hospital), Chongqing, China

**Keywords:** Pancreatic cancer, Senescence-associated secretory phenotype, Tumor microenvironment

## Abstract

**Background:**

Pancreatic cancer (PC) exhibits an extremely poor prognosis due to its high heterogeneity. The senescence-associated secretory phenotype (SASP), a distinct secretory profile displayed by senescent cells, has been increasingly studied. However, the role of SASP in PC prognosis and treatment remains unclear.

**Methods:**

Transcriptomic sequencing data from PC patients were analyzed using consensus clustering based on SASP genes. A prognostic signature was subsequently constructed *via* Least Absolute Shrinkage and Selection Operator (LASSO) regression using survival-related SASP genes. Pathway enrichment analysis for distinct subgroups was performed using Gene Set Variation Analysis (GSVA). Comprehensive analyses of mutational landscapes and tumor immune microenvironments were conducted across risk-stratified PC samples.

**Results:**

Consensus clustering based on SASP genes identified two SASP-associated clusters (SASPclusters), with cluster B demonstrating significantly worse prognosis than cluster A. Thirty-three SASP genes showed significant associations with PC prognosis, and a 7-gene SASP-based prognostic signature was established. High-risk patients exhibited significantly higher mutation rates. Distinct immune cell infiltration patterns, immune functions, checkpoint expression levels, and chemosensitivity profiles were observed between risk groups. Besides, we found that ANGPTL4 could promote PC cell proliferation, migration, and invasion.

**Conclusion:**

Molecular subtyping and risk stratification based on SASP genes effectively predict PC prognosis and reveal heterogeneity in mutational burden, immune microenvironment, and therapeutic sensitivity. These computational findings deepen our understanding of potential role of SASP in PC and provide a theoretical foundation for personalized treatment strategies.

## Introduction

Pancreatic cancer (PC), predominantly pancreatic ductal adenocarcinoma (PDAC), is the most aggressive malignancy of the digestive system, with a five-year survival rate (SR) of ∼12% (Siegel et al., 2023). The pancreas is anatomically concealed; hence, most PC patients are often diagnosed at advanced stages—a phenomenon that is projected to make PC the second deadliest malignancy by 2030 (Nevala-Plagemann, Hidalgo & Garrido-Laguna, 2020; Park, Chawla & O’Reilly, 2021; Rahib et al., 2021). While surgical resection remains the standard intervention for PC, adjuvant therapies, neoadjuvant treatments, immunotherapy, targeted therapy, and radiotherapy (RT) have demonstrated transient efficacy in select populations, although often followed by drug resistance (Mizrahi et al., 2020). Additionally, various factors, including cellular senescence—an emerging focus in cancer research—modulate the tumor microenvironment (TME), highlighting its complex nature, which confers limited response to cytotoxic therapies and immunotherapy in PC patients (Park, Chawla & O’Reilly, 2021).

Cellular senescence is a stress-triggered, irreversible proliferative arrest featuring structural and functional degeneration (Dong et al., 2024; McHugh, Durán & Gil, 2025). Senescence, which was initially recognized as replicative senescence in human fibroblasts, presently encompasses oncogene-induced, therapy-induced, virus-induced, mitochondrial dysfunction-associated, and immune-mediated forms (Wang et al., 2024). Besides growth arrest, senescent cells also exhibit Senescence-Associated Secretory Phenotype (SASP) (Wang et al., 2024), a hyper-secretory profile featuring the secretion of diverse proteins, cytokines, proteases, and chemokines (Dong et al., 2024). According to reports, SASP performs dual roles in tumorigenesis. In addition to inducing cell cycle arrest and immune-mediated damaged cell clearance to suppress tumor initiation, it also fosters immunosuppressive microenvironments conducive to tumor progression and metastasis (Dong et al., 2024; Eggert et al., 2016; Liu & Hornsby, 2007; Orjalo et al., 2009). Furthermore, SASP exerts context-dependent effects in PC. For instance, MEK and CDK4/6 inhibition-induced senescence promoted T-cell infiltration and tumor suppression in murine models (Ohuchida et al., 2004; Ruscetti et al., 2020), whereas radiation-induced fibroblast SASP enhanced PC invasiveness (Ohuchida et al., 2004). Nonetheless, the prognostic implications of specific SASP components in PC remain unclear, necessitating bioinformatics-driven identification of SASP-related prognostic biomarkers.

Herein, we stratified PC patients from the TCGA-ICGC cohort into two SASP subtypes based on the consensus cluster analysis results and established a 7-gene SASP-based prognostic signature. We also conducted functional experiments, validating the role of ANGPTL4 in promoting PC proliferation and metastasis. Our proposed risk stratification model could provide computational evidence to guide prognostic prediction and personalized therapy in PC.

## Materials & Methods

### Data collection

Transcriptomic sequencing and survival data for PC patients were obtained from The Cancer Genome Atlas (TCGA; https://portal.gdc.cancer.gov/) and the International Cancer Genome Consortium (ICGC) (PACA-AU cohort; https://dcc.icgc.org/), with gene expression matrices merged into the combined TCGA-ICGC dataset using the “ComBat” function for batch correction. On the other hand, SASP gene sets were derived from previous literature (Saul et al., 2022), while Copy Number Variation (CNV) files for PC patients were downloaded from UCSC Xena (https://xenabrowser.net/). Additionally, a single-cell RNA sequencing dataset (GSE154778) comprising 10 primary and 6 liver metastatic samples was retrieved from the Gene Expression Omnibus (GEO) database.

### Consensus clustering analysis

We performed consensus clustering utilizing the “ConsensusClusterPlus” R package to explore associations between SASP gene expression and PC subtypes. Survival differences between SASPclusters were compared using Kaplan–Meier (K-M) survival analysis. Gene Set Variation Analysis (GSVA) was performed using all genes from the TCGA-ICGC cohort before comparing differentially enriched pathways between the two SASPclusters and visualizing the results with a heatmap. Differentially Expressed Genes (DEGs) between clusters were identified using the “limma” package (log2FC >1, adjusted *p* < 0.05), followed by functional enrichment analysis.

### Prognostic signature construction and validation

The prognostic value of SASP genes was assessed using Univariate Cox regression, and the prognostic signature was constructed using the Least Absolute Shrinkage and Selection Operator (LASSO) Cox regression. The combined TCGA-ICGC dataset was randomly partitioned into training and validation cohorts (1:1; 1,000 iterations) using the “caret” package. The optimal *λ* value was identified using LASSO regression with 10-fold cross-validation. Genes with non-zero coefficients at the optimal *λ* were selected, from which a 7-gene prognostic signature was constructed, and patients were stratified into low- and high-risk groups. Model performance was evaluated using receiver operating characteristic (ROC) curves generated using the “timeROC” package. Signature gene expressions were visualized using Heatmaps (“pheatmap”), and risk group separation was depicted with the principal component analysis (PCA).

### Clinical relevance of the prognostic signature

Risk score correlations with clinicopathological features (age, sex, pathological grade, tumor nodes metastasis (TNM) stage, and SASPcluster) were analyzed. A nomogram (“rms”) was then developed to predict PC patient survival. Drug sensitivity differences between risk groups were assessed using the “oncoPredict” package, while pathway enrichments across subtypes and risk groups were compared using “GSVA”.

### Mutation analysis

Tumor mutational burden (TMB) was calculated for each patient, with TMB-risk score associations established using Spearman correlation. The risk groups were compared for mutation frequency differences using “maftools”.

### Tumor immune infiltration analysis

Immune cell infiltration levels were quantified using the CIBERSORT algorithm. Immune cell distribution differences between risk groups were visualized using Violin plots. Associations between risk scores and immune cell infiltration were assessed using Spearman correlation analysis with the “psych” package. Immune function activity and checkpoint expression disparities were evaluated using GSVA.

### Single-cell RNA-seq analysis

Single-cell data underwent stringent quality control processes: nFeature_RNA >300 & <6,000, percent.mt <10, percent. HB <1, and nCount_RNA <50,000. Data normalization, variable gene identification (2,000 genes), scaling, and integration were performed using “Seurat” and “harmony”. Dimensionality reduction was visualized using PCA and UMAP. Cell clusters (resolution = 2) were annotated based on marker genes.

### Cell lines

Signature gene protein expression in normal and tumor tissues was validated using the Human Protein Atlas (HPA) (Thul et al., 2017). The MIA PaCa-2 and PANC-1 cell lines (Wuhan Pricella Biotechnology Co., Ltd., Wuhan, China) were cultivated in DMEM culture medium supplemented with 10% Fetal Bovine Serum (FBS; Gibco, Billings, MT, USA) and identified using Short Tandem Repeat (STR).

### Cellular senescence induction

First, H_2_O_2_ was added to the cell culture medium to a final concentration of 50 mM and incubated at 37 °C for 2 h (Purcell, Kruger & Tainsky, 2014). Senescence was then detected using Western Blot (WB) analysis. The primary antibodies included anti-p21 antibody (10355-1-AP, 1:2000; Proteintech, Rosemont, IL, USA), anti-16 antibody (10883-1-AP, 1:3000; Proteintech, Rosemont, IL, USA), and anti-GAPDH antibody (60004-1-lg 1:50000; Proteintech, Rosemont, IL, USA).

### Enzyme-Linked Immunosorbent Assay

Cell culture supernatants (CM) from H_2_O_2_-induced senescent PC and control cells were collected, and secreted protein levels of ANGPTL4 were measured using the ElaBoXTM Human Angiopoietin-like 4 Enzyme-Linked Immunosorbent Assay (ELISA) Kit (SEKH-0091; Solarbio, Beijing, China), with concentrations determined following the manufacturer’s instructions.

### Cell proliferation assay

Cell proliferation was assessed using the Cell Counting Kit 8 (CCK-8) kit (Beyotime Biotechnology, Shanghai, China). The cells (1,000/well) were seeded into 96-well plates and cultured with a recombinant ANGPTL4 protein. Absorbance (Optical Density (OD) 450 nm) was measured daily for 4 days.

### Migration and invasion assays

Migratory and invasive capacities were evaluated using Wound healing and Transwell assays. For the wound healing assay, confluent cells in 6-well plates were scratched with a 200-µl pipette tip and cultured in serum-free medium containing rANGPTL4 (100 ng/ml). For the Transwell assay, cells in serum-free medium (5 × 10^4^) were seeded into Matrigel-coated chambers (Corning, Corning, NY, USA), while complete medium containing rANGPTL4 (100 ng/ml) was added to the lower chamber and incubated for 48 h. Migrated cells were fixed (4% Paraformaldehyde (PFA)) and stained (crystal violet).

### Statistical analysis

Analyses were conducted in R (v4.2.1). Random sampling (“caret”) divided cohorts. Log-rank tests compared Kaplan–Meier survival curves. Spearman correlations assessed associations. Statistical significance thresholds: ***p < 0.001; **p < 0.01; *p < 0.05; ns *p* > 0.05.

## Results

### Genetic alterations of SASP genes

**Figure 1:**
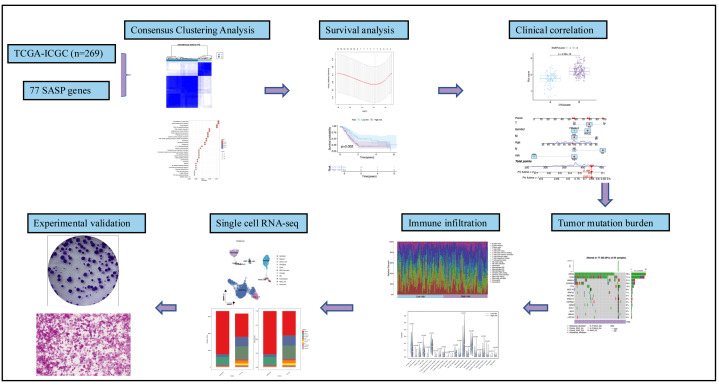
Workflow.

**Figure 2:**
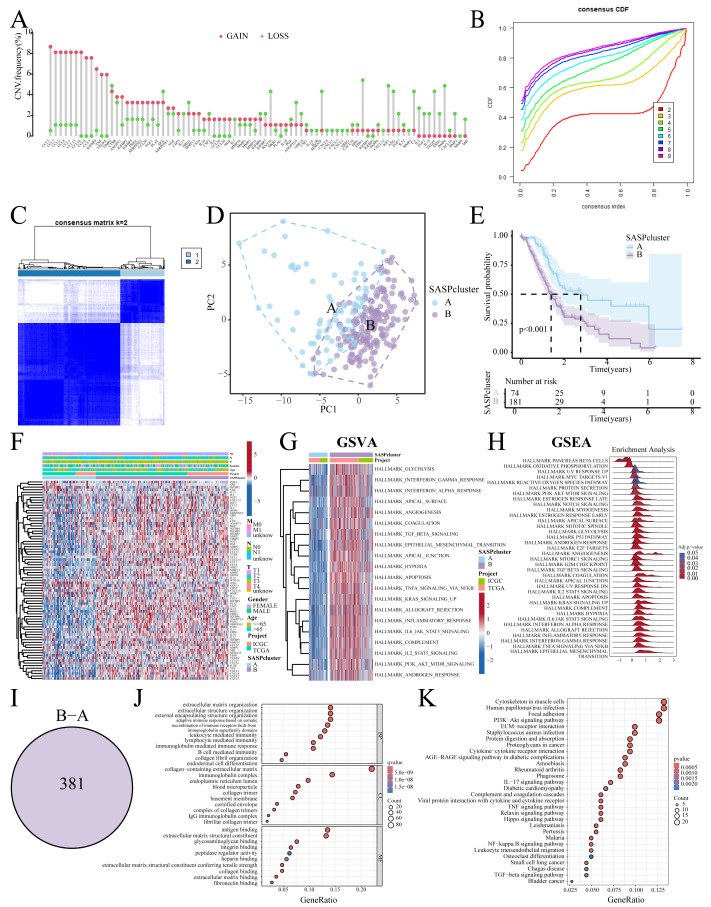
SASP cluster identification. (A) Bar graph showing the CNV frequency of SASP genes; (B, C) Consensus clustering analysis; (D) Sample distribution PCA plot for SASPclusters; (E) Survival plot for SASPclusters; (F) Heatmap depiction of SASP gene expression between SASPclusters; (G, H) GSVA and GSEA results of enrichment pathways between SASPclusters; (I) Venn plot of DEGs between SASPclusters; and (J, K). GO and KEGG bubble plots of DEGs between SASPclusters.

Figure 1 summarizes the workflow, and Table S1 lists the 77 SASP genes. The merged TCGA-ICGC dataset comprised a cohort of 269 PC patients. Somatic CNV analysis revealed frequent alterations: CCL5, CCL7, and CCL2 exhibited copy number gains, while BMP6, VEGFC, and EDN1 showed losses (Fig. 2A).

### Identification of SASP subtypes

The patients were stratified into two SASPclusters (A and B) using consensus clustering (Figs. 2B, 2C), with PCA confirming distinct separations (Fig. 2D). Compared to Cluster A, Cluster B exhibited significantly worse Overall Survival (OS) (Fig. 2E). Heatmaps further revealed elevated SASP gene expression in Cluster B (Fig. 2F), with GSVA also showing the enrichment of the glycolysis, Epithelial-Mesenchymal Transition (EMT), hypoxia, and PI3K-AKT-mTOR pathways in Cluster B (Fig. 2G). A consistent pattern of enrichment was observed in the Gene Set Enrichment Analysis (GSEA, Fig. 2H). Cluster comparisons identified 381 DEGs (Fig. 2I). Gene Ontology (GO) enrichment analysis revealed significant associations with Extracellular Matrix (ECM) organization and integrin binding (Fig. 2J). On the other hand, Kyoto Encyclopedia of Genes and Genomes (KEGG) pathway analysis revealed enrichment in the ECM–receptor interaction, PI3K–Akt signaling, and cytokine–cytokine receptor interaction pathways (Fig. 2K).

### Prognostic signature construction and validation

**Figure 3:**
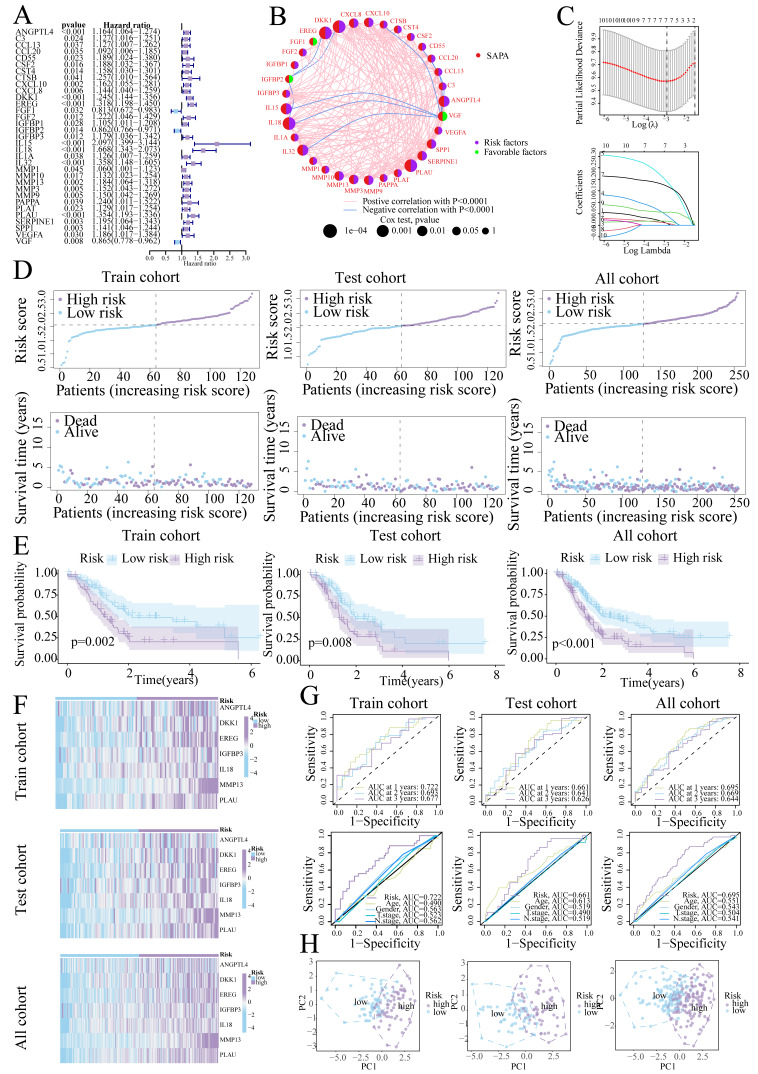
Prognostic signature construction. (A) Univariate Cox regression analysis of the SASP gene. (B) Interaction circle diagram of SASP genes. (C) LASSO regression to construct prognostic models. (D) Distribution of patients at different risks. (E) Survival curves for patients at different risks. (F) Heatmap of signature gene expression in patients with different risks. (G) ROC curves for patients at different risks. (H) PCA distribution of patients with different risks.

Univariate Cox regression identified 33 prognostic SASP genes (Fig. 3A), of which FGF1 (HR = 0.813), IGFBP2 (HR = 0.862), and VGF (HR = 0.865) emerged as protective factors. Protein-Protein Interaction (PPI) networks highlighted gene connectivity (Fig. 3B), with LASSO regression yielding a 7-gene signature: Risk score = (0.0081 × ANGPTL4) + (0.0086 × DKK1) + (0.0177 × EREG) + (0.0425 × IGFBP3) + (0.1902 × IL18) + (0.1702 × MMP13) + (0.0567 × PLAU) (Range: 0.58–3.22, Fig. 3C). Furthermore, high-risk patients exhibited shorter survival (Figs. 3D, 3E). High-risk patients also exhibited signature gene upregulation (Fig. 3F). The AUC values for the training cohort were 0.722 (1 year), 0.693 (2 years), and 0.677 (3 years); for the test cohort, they were 0.661 (1 year), 0.641 (2 years), and 0.626 (3 years). The overall cohort AUC values were 0.695 (1 year), 0.669 (2 years), and 0.644 (3 years). Compared to traditional clinical characteristics, the risk score demonstrated superior predictive performance in forecasting prognosis for patients with PC (Fig. 3G). The PCA plot demonstrates a clear separation between the two risk groups of patients (Fig. 3H).

### Validation of signature genes in single-cell RNA-seq data

**Figure 4:**
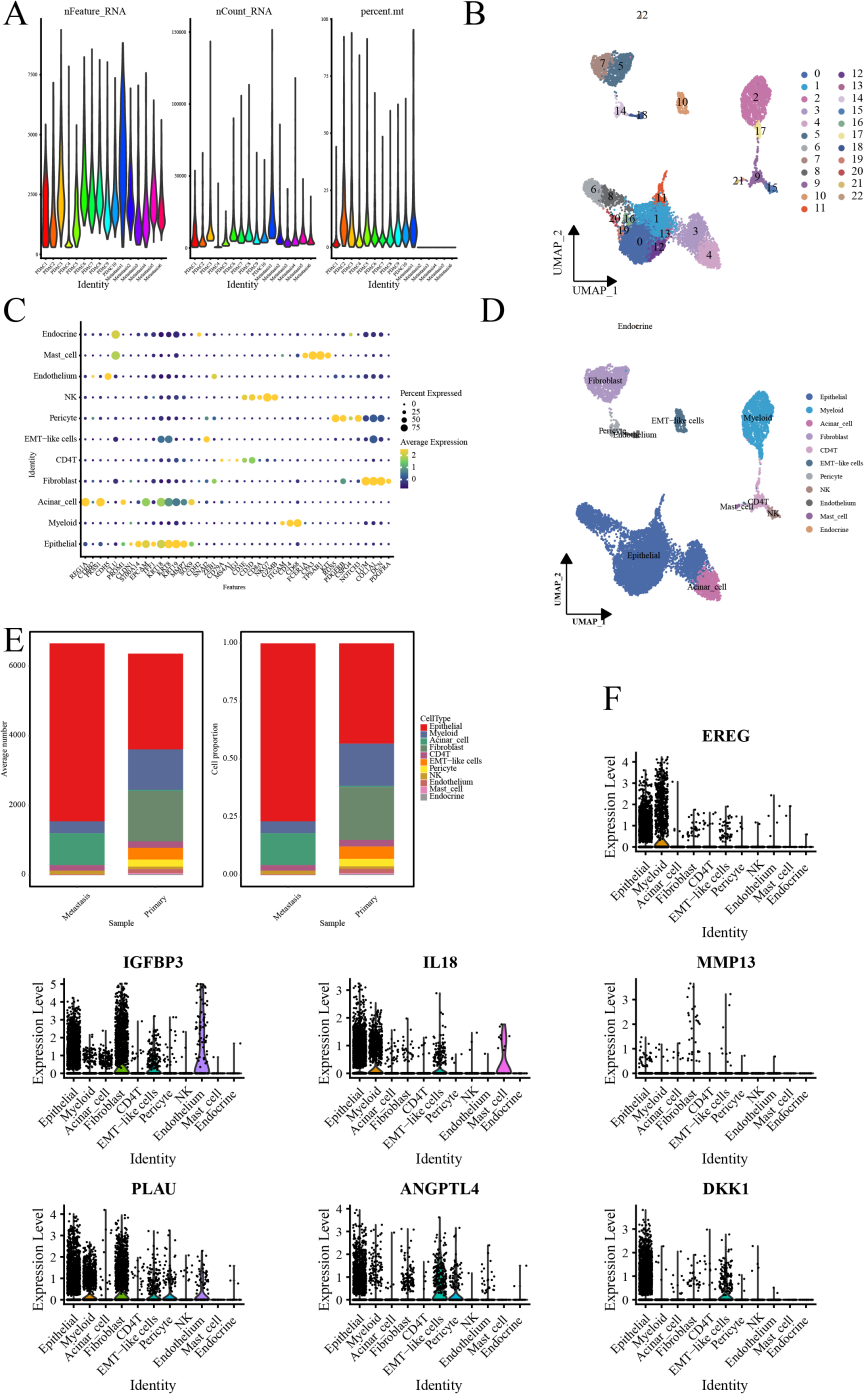
Single-cell RNA-seq data analysis. (A) Violin representation of each sample’s data characteristics; (B) UMPA plots for cell clustering; (C) Bubble plot of cell marker genes for each cell type; (D) UMPA plots for cell types; (E) Bar graph showing the proportion of each cell type; and (F) Violin representation of the expression level of the signature genes in each cell.

Quality control processes yielded 23 cell clusters (Figs. 4A, 4B). Furthermore, annotation revealed 11 cell types, with elevated ductal epithelial cells and fibroblasts in metastatic samples and primary tumors, respectively (Figs. 4C–4E). The signature genes were predominantly expressed in ductal epithelial and myeloid cells (Fig. 4F).

### Clinical relevance of the prognostic signature

**Figure 5:**
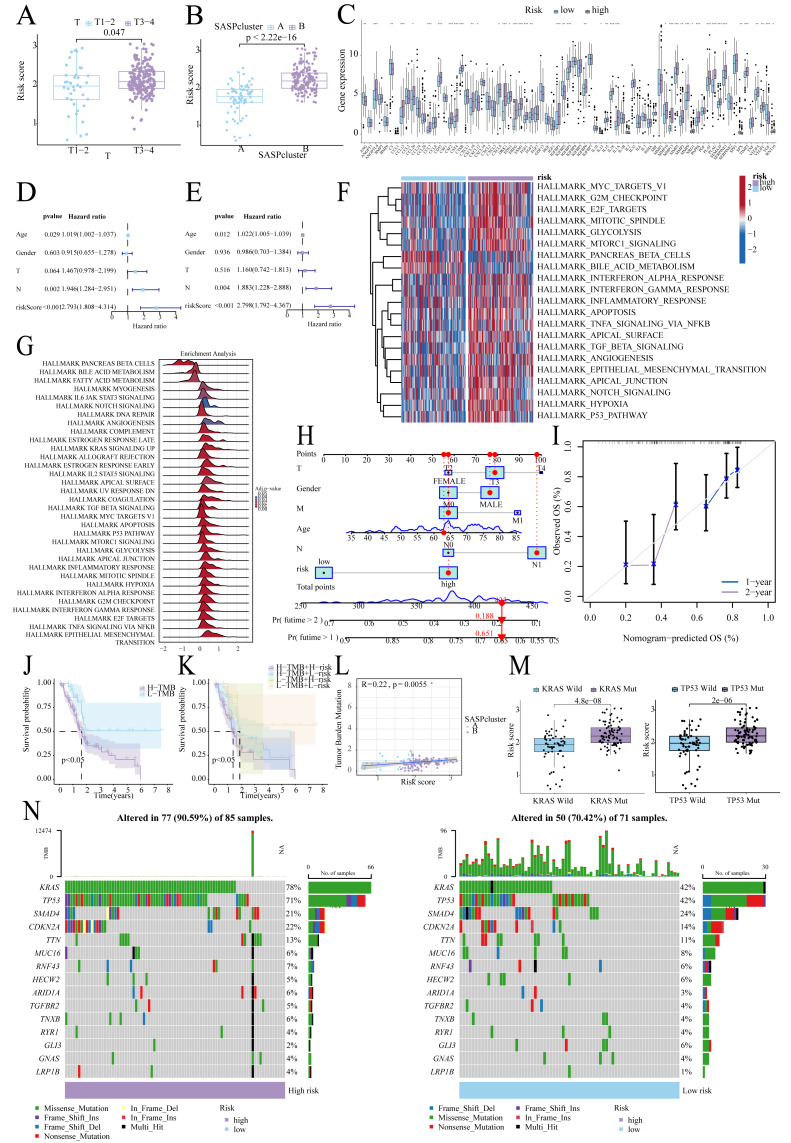
Risk score-clinical characteristics relationship. (A) Relationship between risk score and T stage; (B) Relationship between risk score and SASPcluster; (C) SASP gene expression across patients with different risk levels; (D, E). Univariate and multivariate Cox regression analysis; (F, G) GSVA and GSEA results showing pathway enrichment between patients with different risk levels; (H, I). Nomogram construction and validation; (J, K). Survival curves for TMB and risk score; (L) Scatterplot of TMB and SASPcluster correlations; (M) Scatterplot of risk score with KRAS and TP53 mutation frequency; and (N) Waterfall plot of risk score with gene mutations in PDAC samples.

Higher risk scores correlated with advanced T-stage (Fig. 5A) and SASPcluster B (Fig. 5B), highlighting the significance of SASP in tumor development or risk. Furthermore, most SASP genes showed differential expression between groups (Fig. 5C). Multivariate Cox regression confirmed risk score as an independent prognostic factor (Figs. 5D, 5E), and GSVA and GSEA highlighted the enrichment of the glycolysis, mTORC1, and EMT pathways in high-risk patients (Figs. 5F, 5G). Moreover, the resulting nomogram effectively predicted survival (Figs. 5H, 5I).

### Tumor mutational landscape

Low-TMB patients, particularly in the low-risk subgroup, exhibited better survival (Figs. 5J, 5K), and TMB correlated positively with risk scores (Fig. 5L). Furthermore, KRAS/TP53 mutations correlated with higher risk scores (Fig. 5M), and high-risk patients demonstrated greater mutation frequencies (90.59% *vs.* 70.42%; Fig. 5N).

### Association of prognostic signature with TME

**Figure 6:**
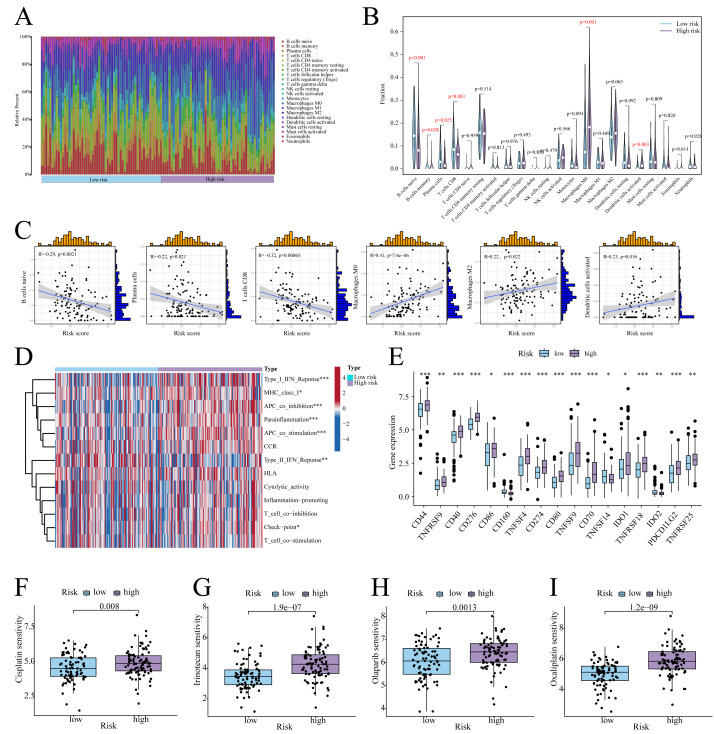
Immune infiltration analysis. (A) Histogram of the level of immune cell infiltration. (B) Violin plot of the level of immune cell infiltration. (C) Scatterplot of the correlation between the level of immune cell infiltration and risk score. (D) GSVA heatmap of immune function in patients at different risks. (E) Violin plots of immune checkpoint expression in patients at different risks. (F–I) Sensitivity to commonly used chemotherapeutic agents in patients at different risks. ^∗^ indicates *p* < 0.05, ^∗∗^ indicates *p* < 0.01, ^∗∗∗^ indicates *p* < 0.001.

According to CIBERSORT analysis, high-risk patients exhibited naive B cell, memory B cell, plasma cell, and CD8+ T cell downregulation, alongside M0 macrophage and activated Dendritic Cell (DC) upregulation (Figs. 6A, 6B). The risk score exhibited a negative correlation with naïve B cells, plasma cells, and CD8 T cells, whereas it showed a positive correlation with Macrophage M0, Macrophage M2, and activated dendritic cells (Fig. 6C). Furthermore, in high-risk patients, immune functions (*e.g.*, Type I IFN response, checkpoint activity) were suppressed (Fig. 6D), whereas checkpoints CD44, PD-L1, and PDCD1LG2 were upregulated (Fig. 6E). On the other hand, low-risk patients showed enhanced sensitivity to cisplatin, irinotecan, olaparib, and oxaliplatin (Figs. 6F–6I).

### Functional validation of ANGPTL4 in PC

**Figure 7:**
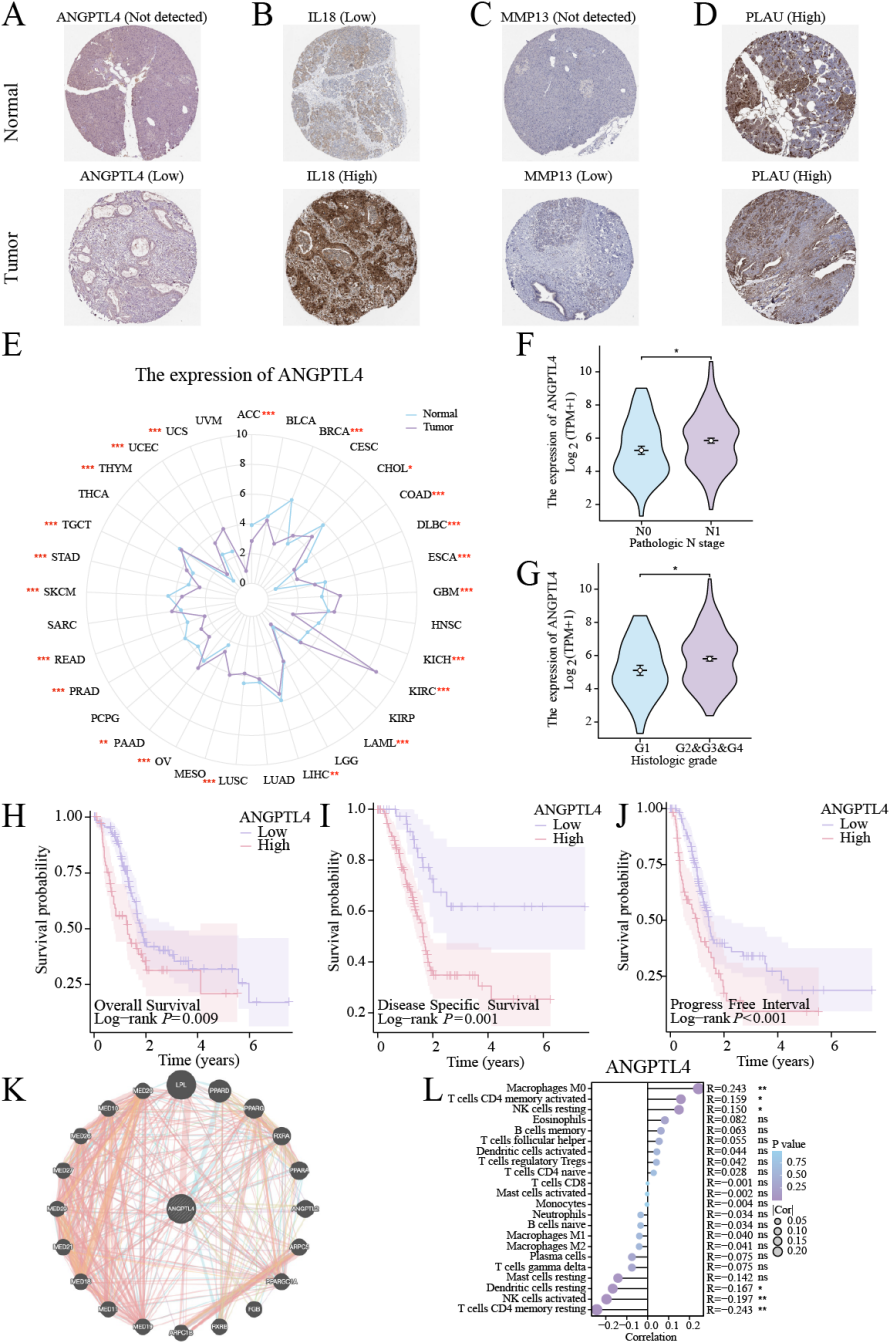
Analysis of ANGPTL4: (A–D) IHC image of the signature gene in the HPA database. (E) Radar plot of ANGPTL4 expression levels in pan-cancer. (F, G) Relationship between ANGPTL4 with tumor differentiation and PC lymph node metastasis. (H–J) Survival curves of ANGPTL4 in PC tissues. (K) Circle diagram of genes interacting with ANGPTL4. (L) Correlation of ANGPTL4 expression with the level of PC tissue immune cell infiltration. ^∗^ indicates *p* < 0.05, ^∗∗^ indicates *p* < 0.01, ^∗∗∗^ indicates *p* < 0.001.

**Figure 8:**
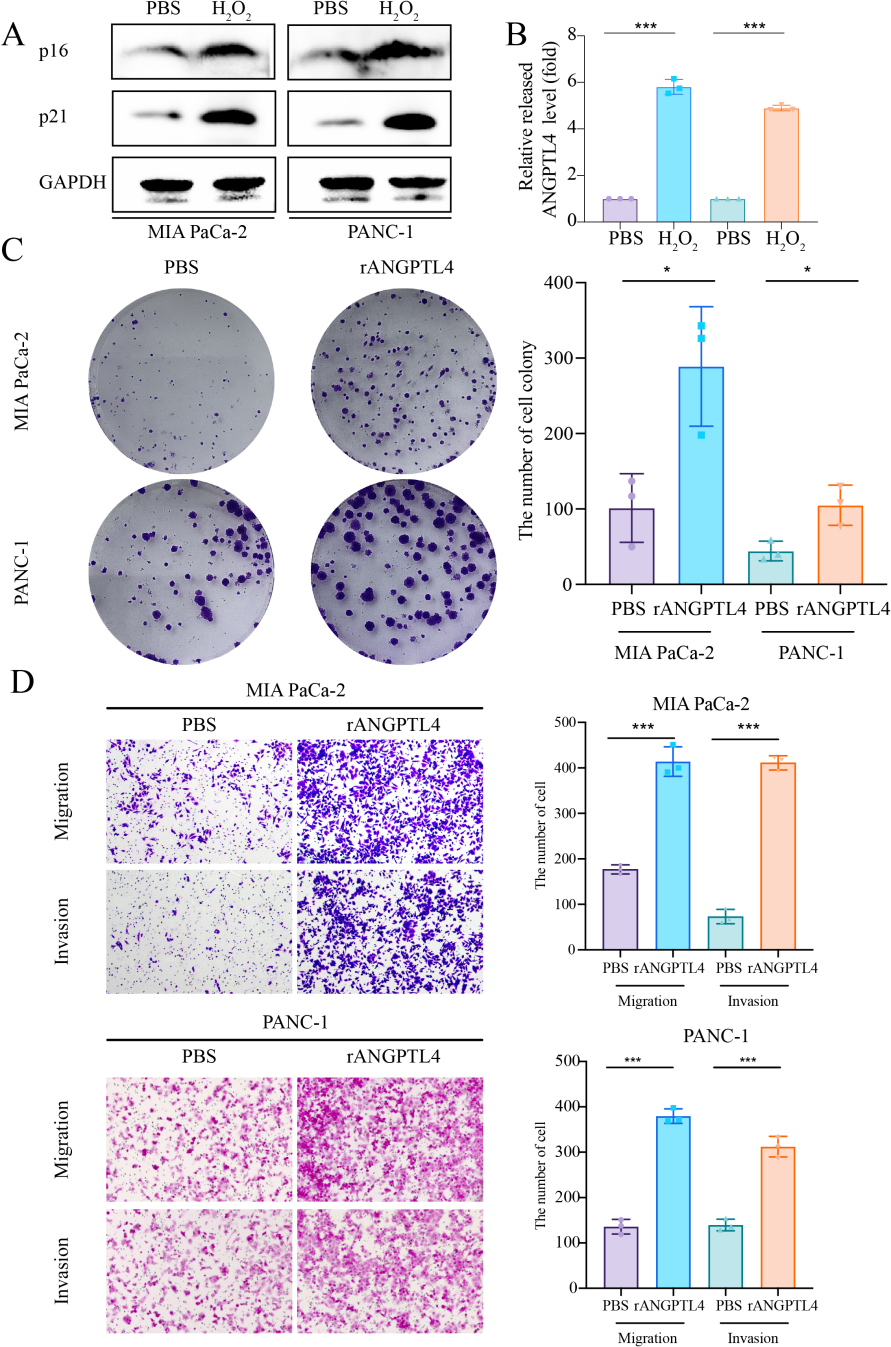
Role of ANGPTL4 on PC proliferation and migration. (A) Western blot detection of p21 and p16 to validate the cell senescence model. (B) ELISA for detecting ANGPTL4 levels secreted by senescent cells. (C) Clone formation results of rANGPTL4 promoting PC cell proliferation. (D) Transwell results of rANGPTL4 promoting PC cell migration and invasion (20x). ^∗^ indicates *p* < 0.05, ^∗∗^ indicates *p* < 0.01, ^∗∗∗^ indicates *p* < 0.001.

Centrosome amplification-induced senescence-associated secretory phenotype variation activated the Hypoxia-Inducible Factor-1*α* (HIF-1*α*) and related gene pathways, including migration-promoting factors such as ANGPTL4, highlighting the significance of ANGPTL4 in SASP (Wu et al., 2023). Herein, immunohistochemistry (IHC) analysis revealed ANGPTL4, IL-18, MMP13, and PLAU upregulation in PC tissues (Figs. 7A–7D). Furthermore, pan-cancer analysis revealed ANGPTL4 overexpression in PC, as well as breast, colorectal, and liver cancers (Fig. 7E). High ANGPTL4 expression correlated with a poor prognosis, poor differentiation, and lymph node metastasis (LNM) (Figs. 7F–7J). Additionally, GeneMANIA identified the interactions of ANGPTL4 with LPL, PPARD, and PPARG (Fig. 7K), with ANGPTL4 expression correlating positively with M0 macrophages, and negatively with NK cells activated and T cells CD4+ memory resting (Fig. 7L). We also detected the cellular senescence markers p21 and p16 to validate the construction of the oxidative stress (OS)-induced cellular senescence model (Fig. 8A). According to the ELISA results, senescent cells secreted significantly higher levels of ANGPTL4 compared to the control group, confirming ANGPTL4’s involvement in SASP (Fig. 8B). Functional assays further demonstrated rANGPTL4-driven cell proliferation (Fig. 8C), migration, and invasion (Fig. 8D).

## Discussion

The global annual PC diagnosis has doubled over the past two decades, posing substantial challenges to public health systems and society as a whole (Klein, 2021). Furthermore, effective treatments for PC remain limited. Although immunotherapy and targeted therapies demonstrated great promise, the poor response attributed to the unique immunosuppressive TME of PC has introduced new challenges.

Cellular senescence, a biological concept first proposed in 1961, has extended beyond replicative exhaustion to include stress-induced senescence (Dong et al., 2024). Tumor heterogeneity dictates that senescence adopts the “double-edged sword” adage in cancer progression. While senescence restricts uncontrolled proliferation *via* cell cycle arrest or immune cell recruitment, SASP secretes factors that shape an immunosuppressive TME, thus promoting tumor progression (Dong et al., 2024; McHugh, Durán & Gil, 2025). Despite extensive research on senescence and SASP, their roles in PC remain unclear, forming the basis of this study, which aimed to explore the associations of SASP-related genes with PC molecular subtypes, prognosis, genetic mutations, TME infiltration, and chemosensitivity.

Our findings revealed two SASP-associated clusters (SASPclusters) based on SASP gene expression. Compared to Cluster A, Cluster B exhibited worse prognosis and higher SASP gene expression. Furthermore, a 7-gene prognostic model was constructed using LASSO regression, effectively stratifying the patients into high- and low-risk groups. High-risk patients exhibited signature gene overexpression, predominantly localized to epithelial and myeloid cells in single-cell RNA-seq analyses. Additionally, cancer-associated pathways, including glycolysis, angiogenesis, and EMT, were enriched in Cluster B and high-risk patients. Nacarelli et al. (2019) reported that the HMGA-NAMPT-NAD+ axis could enhance glycolysis and mitochondrial respiration, driving pro-inflammatory SASP. Furthermore, in preclinical PDAC models, therapy-induced senescence promoted angiogenesis *via* SASP-secreted factors, improving gemcitabine efficacy (Ruscetti et al., 2020). Similarly, senescent fibroblast-secreted SASP promoted EMT in premalignant epithelia (Yang et al., 2018), while myofibroblast-derived SASP facilitated EMT in oral submucous fibrosis (OSF) (Sharma et al., 2021), implying shared mechanisms between Cluster B and high-risk patients.

Chemotherapy and radiotherapy (RT) eliminate cancer cells, while making them, along with normal cells, senescent. Although cellular senescence could inhibit tumor progression, residual senescent cells post-treatment may pose new risks, such as promoting cancer stem cell (CSC) activation and re-proliferation, ultimately causing tumor recurrence (Nardella et al., 2011; Milanovic et al., 2018). Besides alleviating treatment-related side effects, combining senescent cell clearance with existing cancer therapies might also significantly reduce the risk of tumor progression (Xiao et al., 2023). Navitoclax (ABT263) could inhibit the activity of anti-apoptotic proteins, clearing senescent cells. It could also effectively eliminate PARP inhibitor olaparib-induced senescent ovarian and breast cancer cells (Fleury et al., 2019). Notably, navitoclax-galactose (Nav-Gal) could be a potent senescent cell clearance intervention, selectively inducing senescent cell apoptosis, with a senescent cell clearance index higher than that of navitoclax alone. It enhanced the cytotoxic effect of cisplatin in A549 lung cancer (LC) cells. Additionally, Cisplatin+Nav-Gal effectively eliminated senescent LC cells and significantly inhibited tumor growth (González-Gualda et al., 2020). Furthermore, artemisinin could inhibit irinotecan treatment-activated mTOR activity, alleviating irinotecan treatment-induced intestinal senescence. Moreover, drug combinations significantly inhibited colorectal tumor growth in mice (Jia et al., 2023). A combination formulation comprising ch282-5 and chloroquine, rapamycin, oxaliplatin, or navitoclax effectively inhibited colon cancer growth and liver metastasis (Wang et al., 2016). Overall, in addition to effectively inhibiting tumor progression, the combined use of chemotherapy drugs and the senescence-inducing agent navitoclax may improve high-risk patients’ low sensitivity to drugs such as olaparib, cisplatin, irinotecan, and oxaliplatin, providing novel insights for clinical treatment.

Herein, the established model comprised several signature genes: IL18, MMP13, PLAU, IGFBP3, EREG, DKK1, and ANGPTL4. Interleukin-18 (IL-18) could downregulate the anti-aging protein Klotho, promoting fibroblast senescence in pulmonary fibrosis (Zhang et al., 2019a). In tumors, IL-18/IL-12-stimulated *γδ* T cells directly kill cancer cells and induce senescence in the absence of TCR signaling, exerting potent anti-tumor activity (Schilbach et al., 2020). However, IL-18 is also recognized as a tumor-promoting factor (Zhang et al., 2024). This duality mirrors SASP’s context-dependent roles, where IL-18 exerts contrary effects under various conditions. During EMT, Matrix Metallopeptidase 13 (MMP13), a key regulator of chondrocyte senescence in Osteoarthritis (OA) (Zhang et al., 2019b), is upregulated, contributing to PC metastasis (Rath et al., 2017; Wei et al., 2017). However, its role in PC cell senescence remains unclear. According to reports, hyperglycemia might promote endothelial senescence *via* the AQR/PLAU axis (Wan et al., 2022). Senescent cancer-associated fibroblasts (CAFs) secrete plasminogen activator urokinase (PLAU), driving PC progression and immunosuppression (Liu et al., 2025). Additionally, PLAU induces neuronal senescence (Sun et al., 2024), highlighting its dual role as an outcome and inducer of SASP. Although senescent Endometrial Mesenchymal Stromal Cell (EMSC)-secreted Insulin-like Growth Factor-Binding Protein 3 (IGFBP3) (Ushakov et al., 2021) could induce senescence *via* telomerase inhibition, thus suppressing BC proliferation (Kwon et al., 2023), its role in PC senescence remains unclear. On the other hand, Epiregulin (EREG), a senescent stromal cell-secreted soluble factor, was identified as a potential therapeutic target for SASP and could be leveraged to overcome treatment resistance (Wang et al., 2022a; Wang et al., 2022b). While EREG upregulation in PC tissues could stimulate tumor growth, its silencing inactivates ERK/p38 MAPK pathways, thus inhibiting PDAC (Liu et al., 2024; Zhu et al., 2000). Nonetheless, the relationship between EREG and PC senescence remains undefined. Regarding DKK1, its knockout in adipose-derived stem cells was reported to activate Wnt signaling, enhancing cell viability, reducing senescence, and suppressing NF-*κ*B-mediated inflammation (Choi et al., 2024). Exogenous DKK1 partially rescued FEZ1 inhibition-induced growth arrest and senescence in limbal stem/progenitor cells (Zhu et al., 2023). Furthermore, YAP prevented dermal fibroblast senescence and inhibited melanogenesis *via* DKK1 paracrine signaling (Li et al., 2024). Additionally, ZEB1 activated the DKK1/mutant p53/Mdm2/CtBP axis and suppressed macroH2A1-mediated senescence, promoting tumorigenesis (De Barrios et al., 2017). Finally, ANGPTL4, a key mediator of senescence in diabetic cardiomyopathy, exhibits dual roles. Centrosome amplification-induced SASP promoted tumorigenesis *via* ANGPTL4 secretion (Wen et al., 2024). Furthermore, ANGPTL4 accelerated KRAS(G12D)-driven acinar-to-ductal metaplasia and PC initiation (Yan et al., 2021). Its overexpression also induced pro-invasive, proliferative, and anti-apoptotic transcriptional programs, conferring gemcitabine resistance and shortening survival in PC (Gordon et al., 2023). Herein, bioinformatics analyses revealed ANGPTL4 expression in epithelial, EMT-like, and perivascular cells, correlating with PC prognosis. Functional experiments further demonstrated that recombinant ANGPTL4 could promote PC cell proliferation, invasion, and migration, although its link to senescence remains unclear, necessitating further investigation.

Herein, high-risk patients exhibited reduced naive B cell, memory B cell, plasma cell, and CD8+ T cell infiltration. While B cells amplify anti-tumor immunity *via* antibody production, the presence of plasma cells has been associated with favorable immunotherapy responses (Delvecchio et al., 2022; Mirlekar et al., 2022). Additionally, IL-35/STAT3/PAX5/BCL6 signaling-driven transcriptional dysregulation in naïve B cells could impair their differentiation into anti-tumor effector cells (Mirlekar et al., 2022). Fan et al. (2024) reported that DKK1+ tumor cells suppressed CCL19+ fibroblasts and plasma cell infiltration, contributing to immunotherapy resistance in hepatocellular carcinoma (HCC). Elevated DKK1 levels and reduced plasma cell infiltration in high-risk PC patients suggest similar interactions, warranting further validation. Furthermore, PC patients often exhibit suppressed CD8+ T cell infiltration, which is critical for tumor control. Although MEK/CDK4/6 inhibition-induced SASP enhanced CD8+ T cell infiltration and PD-1 inhibitor sensitivity in PDAC models (Ruscetti et al., 2020), our findings of CD8+ T cell downregulation in high-risk patients suggest context-dependent mechanisms. Therefore, given the higher KRAS mutation rates in high-risk patients, combining KRAS pathway inhibitors with PD-1 blockade may enhance CD8+ T cell infiltration, improving outcomes. Primary human senescent CD8+ T cells also exhibit a SASP comprising chemokines, cytokines, and ECM remodeling enzymes (Callender et al., 2018). Furthermore, aging cells could express immune surveillance inhibitors and release ligands for the NKG2D receptor, suppressing their clearance, thus weakening the surveillance of aging cells by NK and cytotoxic CD8+ T cells (Salminen, 2021). In an age-related inflammation model, cells highly expressing PD-L1 were associated with higher levels of SASP, suggesting that senescent cells specifically express PD-L1 to evade monitoring by CD8+ T cells (Wang et al., 2022a; Wang et al., 2022b). Moreover, combinations of KRAS inhibitors could induce RB protein-mediated senescence, inhibiting PDAC proliferation. This senescence-inducing therapy triggers SASP, including pro-angiogenic factors that promote tumor angiogenesis. It is also noteworthy that SASP-mediated Endothelial Cell (EC) activation could stimulate CD8+ T cells to aggregate toward immunologically “cold” tumors, making them sensitive to PD-1 checkpoint blockade (Ruscetti et al., 2020). Consequently, SASP and immune cells might correlate closely. Overall, age-related diseases may evade surveillance by CD8 T cells and others *via* SASP, while immunotherapy targeting tumors may leverage SASP to reshape the immune IME and achieve antitumor therapeutic effects.

Despite its valuable insights, this study has some limitations. First, we mainly derived SASP genes from published literature, potentially omitting uncharacterized genes. Second, the mechanism linking the seven marker genes in PC with SASP should be verified further in experimental studies. Third, our prognostic model was constructed and validated based solely on public databases. To further validate the reliability of our prognostic signature, additional multicenter clinical cohort studies will be required in the future.

## Conclusions

Herein, we developed a novel SASP-associated molecular subtype and prognostic signature, which may aid in predicting PC prognosis, potentially illuminating PC stratification and therapeutic strategies. Although high-risk patients identified with the model might benefit from combined KRAS pathway inhibition and immunotherapy, further rigorous experimental studies will be required to validate this phenomenon.

##  Supplemental Information

10.7717/peerj.20476/supp-1 Supplemental Information 1 The gene lists of 77 SASP genes

10.7717/peerj.20476/supp-2 Supplemental Information 2 Code

10.7717/peerj.20476/supp-3 Supplemental Information 3 Raw data The raw data in PZFX format was created using GraphPad Prism (Version 10), downloaded from the official website ( https://www.graphpad.com/features).

## Additional Information and Declarations

### Data Availability

The following information was supplied regarding data availability: The raw data and code is available in the Supplemental Files. The data is available at FigShare: Xiang, Zhiyong; Kuang, Yuewen; Jia, Mingkun; Zhu, Yuming (2025). Analysis of Molecular Subtypes and Prognostic Signature of Senescence-Associated Secretory Phenotype in Pancreatic Cancer. figshare. Dataset. https://doi.org/10.6084/m9.figshare.30621227.v1.
