# Supplementary material for: Analysis of molecular subtypes and prognostic signature of senescence-associated secretory phenotype in pancreatic cancer
Source: PeerJ. 2026 Jan 6;14:e20476. doi: 10.7717/peerj.20476 (PMC12786131; doi:10.7717/peerj.20476)
Supplement: Supplemental Information 1 [file peerj-14-20476-s001.docx]

gene

ANG

ANGPT1

ANGPTL4

BMP2

BMP6

C3

CCL1

CCL13

CCL2

CCL20

CCL24

CCL26

CCL7

CCL8

CD55

CSF1

CSF2

CST4

CTSB

CXCL1

CXCL10

CXCL12

CXCL16

CXCL2

CXCL3

CXCL8

DKK1

EDN1

EREG

ESM1

FGF1

FGF2

FGF7

GDF15

HGF

IGF1

IGFBP1

IGFBP2

IGFBP3

IGFBP4

IGFBP5

IGFBP6

IGFBP7

IL10

IL13

IL15

IL18

IL1A

IL1B

IL2

IL32

IL6

IL7

INHA

MIF

MMP1

MMP10

MMP13

MMP2

MMP3

MMP9

NRG1

PAPPA

PGF

PLAT

PLAU

SEMA3F

SERPINE1

SERPINE2

SPP1

SPX

TIMP2

TNF

VEGFA

VEGFC

VGF

WNT16
