# Supplementary material for: Analysis of molecular subtypes and prognostic signature of senescence-associated secretory phenotype in pancreatic cancer
Source: PeerJ. 2026 Jan 6;14:e20476. doi: 10.7717/peerj.20476 (PMC12786131; doi:10.7717/peerj.20476)
Supplement: Supplemental Information 3 — The raw data in PZFX format was created using GraphPad Prism (Version 10), downloaded from the official website ( https://www.graphpad.com/features). [file peerj-14-20476-s003.zip › Raw data/Raw data.docx]

clone formation

1st

MIA PaCa-2

PBS rANGPTL4


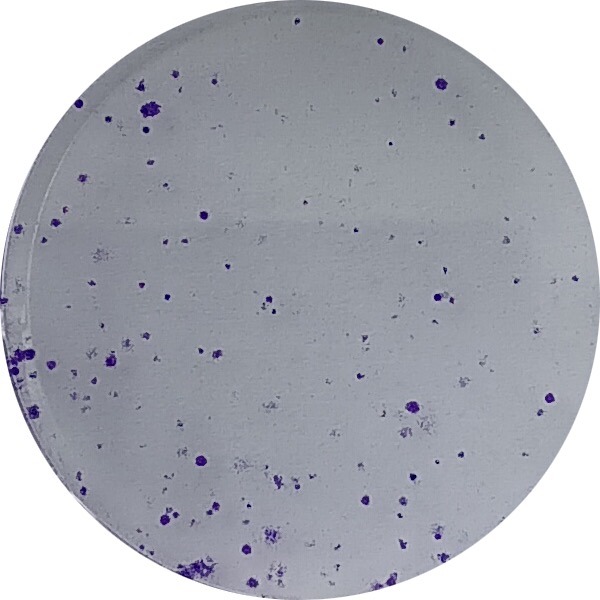

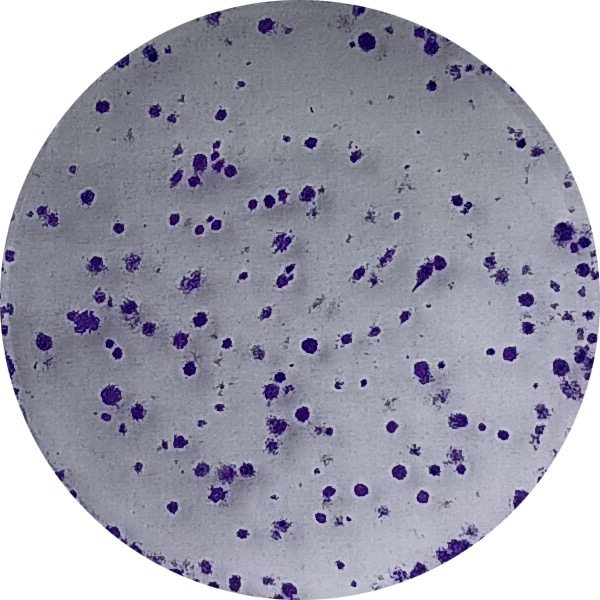


PANC-1

PBS rANGPTL4


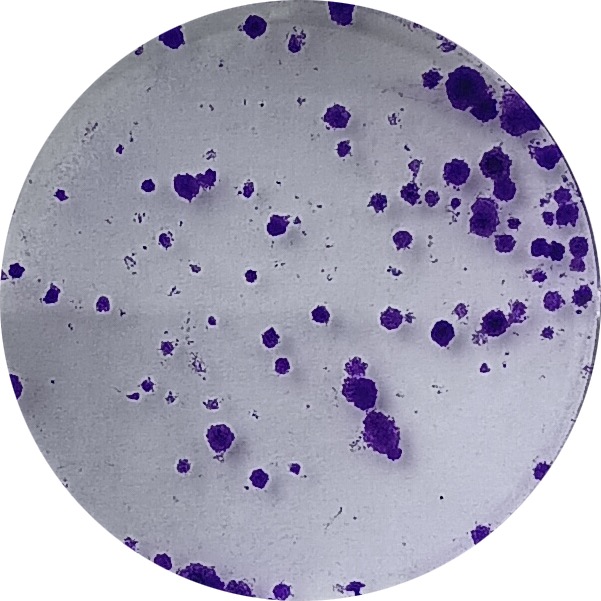

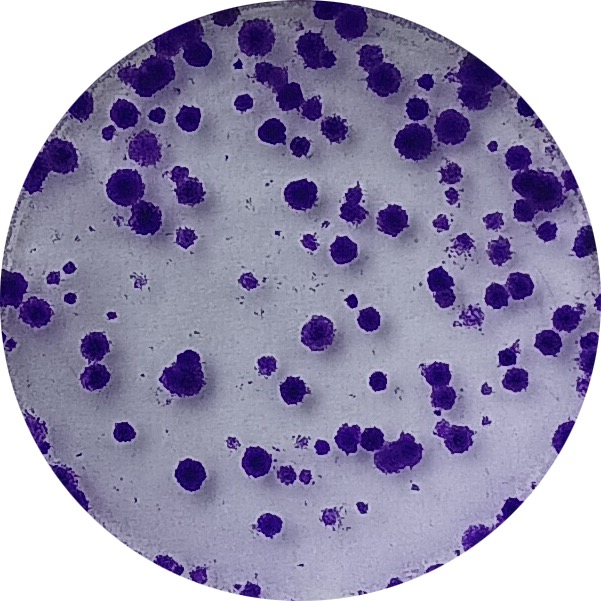


2nd

MIA PaCa-2

PBS rANGPTL4


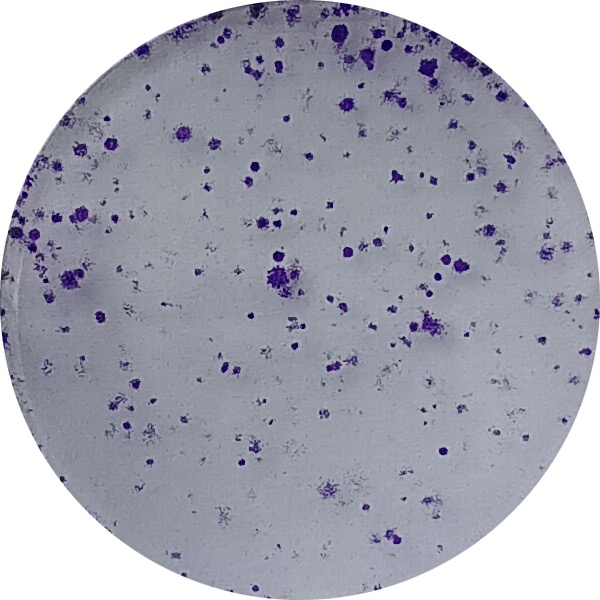

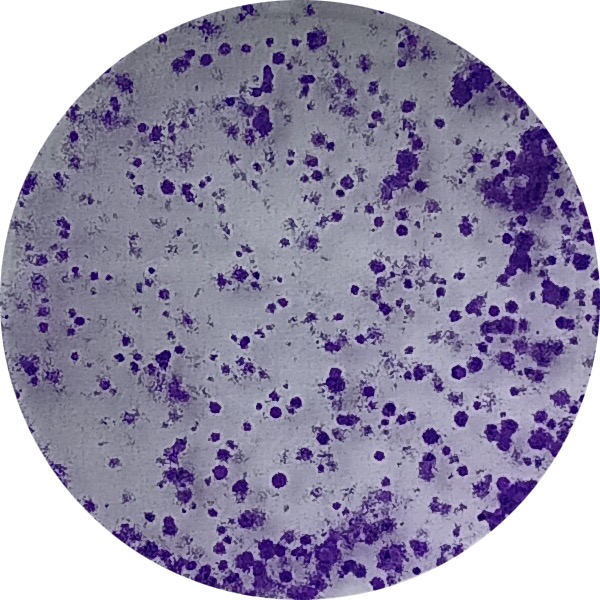


PANC-1

PBS rANGPTL4


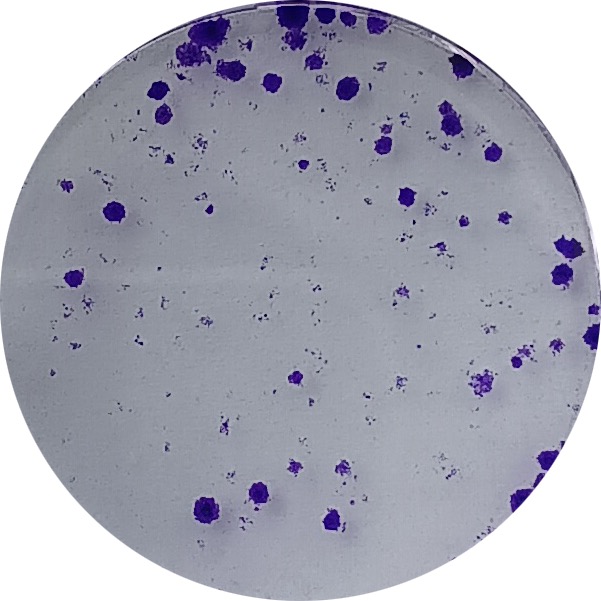

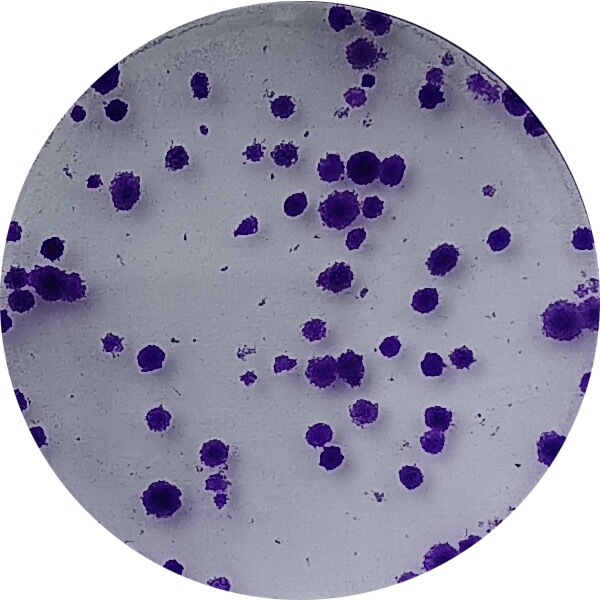


3rd

MIA PaCa-2

PBS rANGPTL4


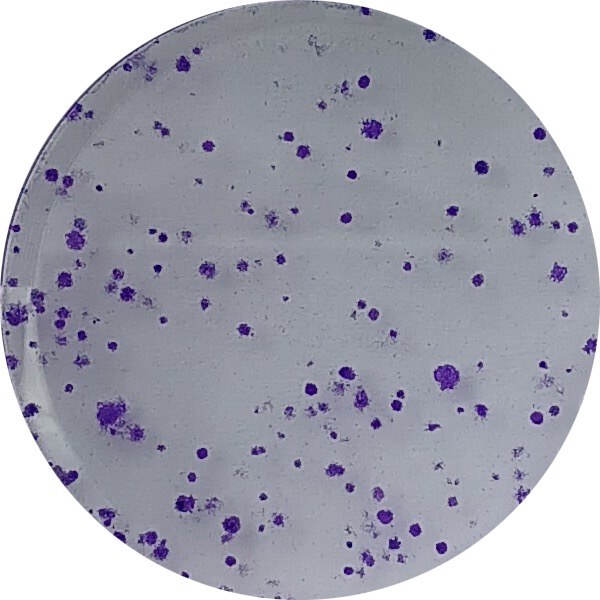

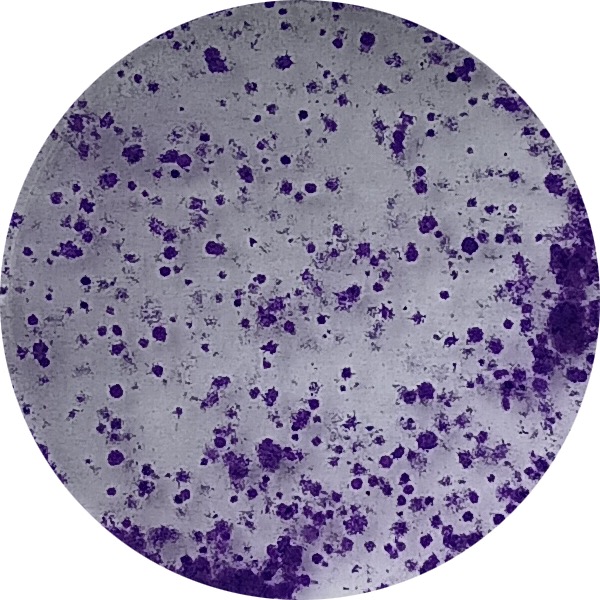


PANC-1

PBS rANGPTL4


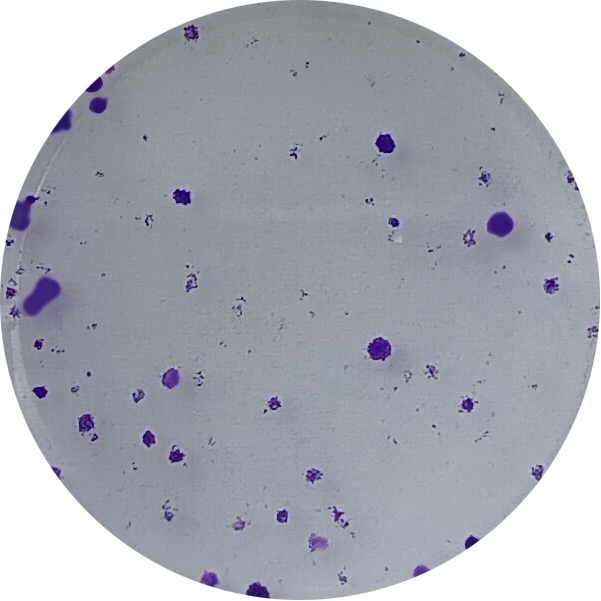

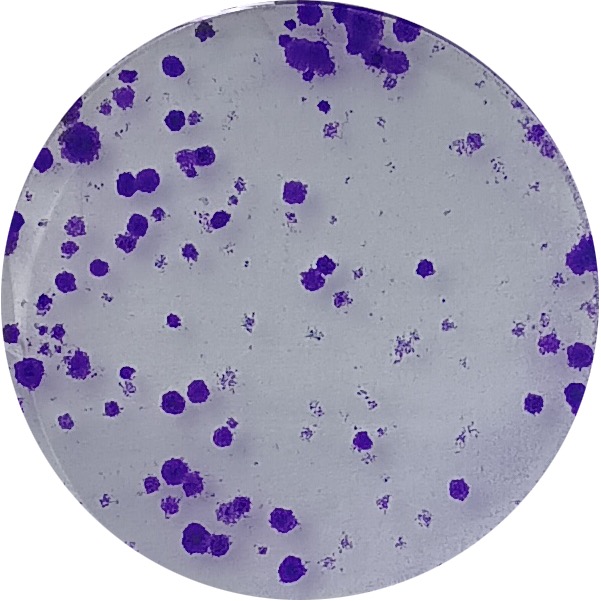


Transwell-migration

1st

MIA PaCa-2

PBS rANGPTL4


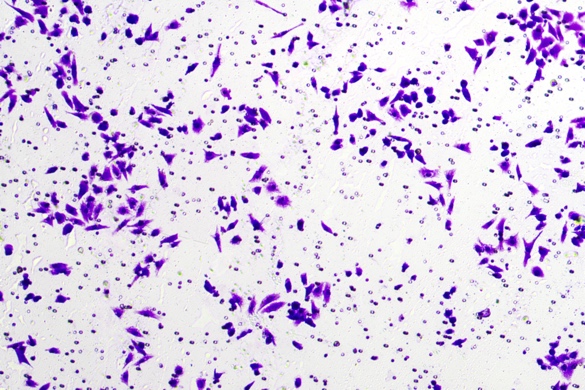

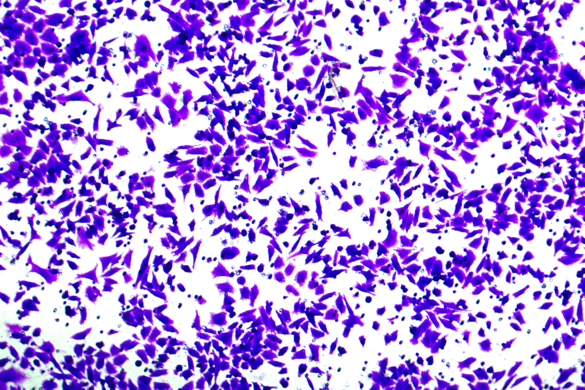


PANC-1

PBS rANGPTL4


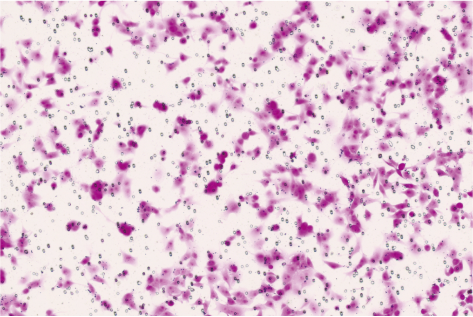

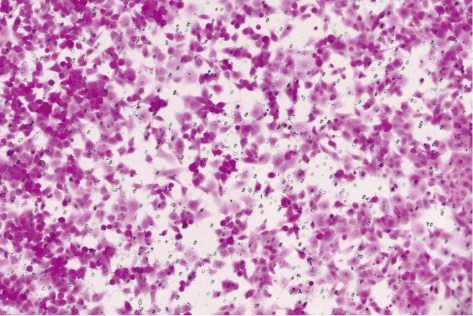


2nd

MIA PaCa-2

PBS rANGPTL4


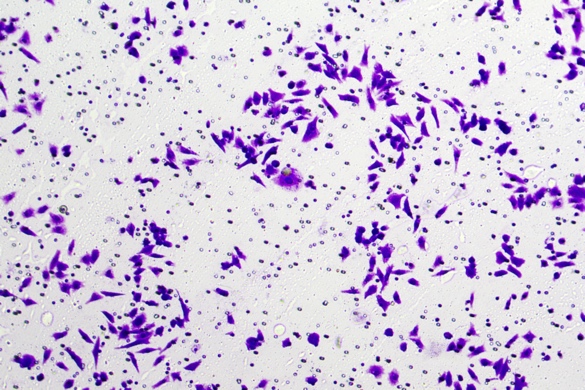

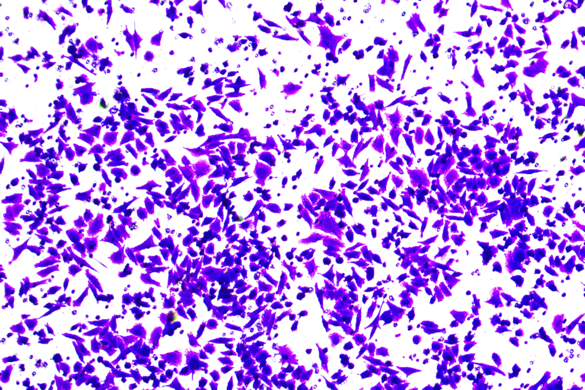


PANC-1

PBS rANGPTL4


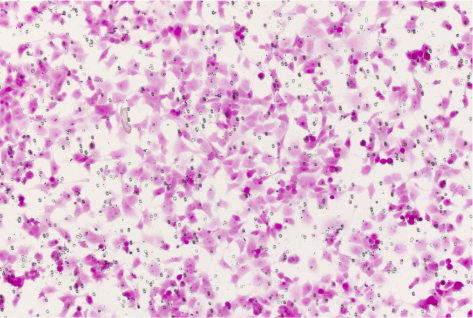

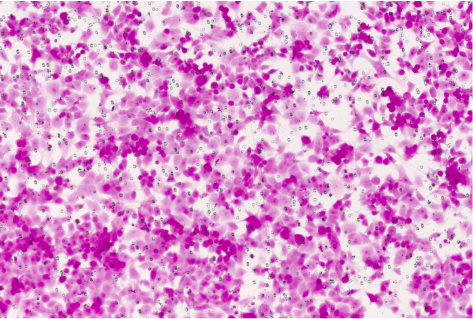


3rd

MIA PaCa-2

PBS rANGPTL4


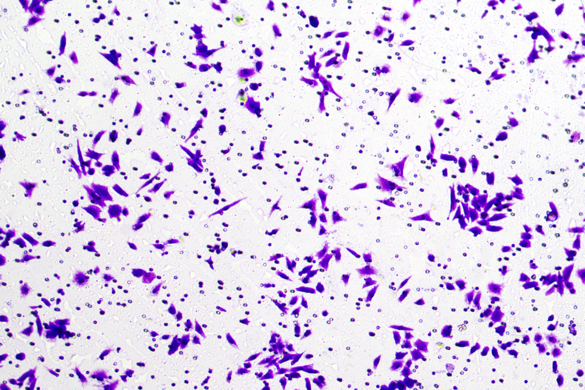

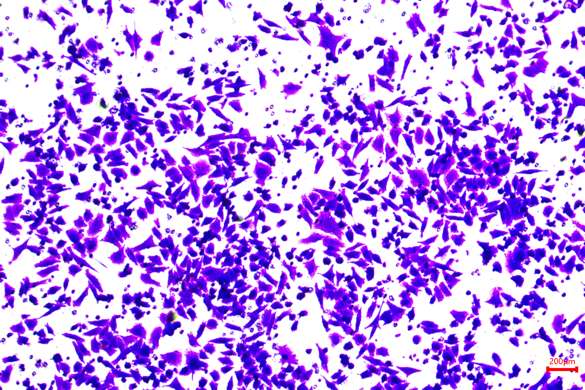


PANC-1

PBS rANGPTL4


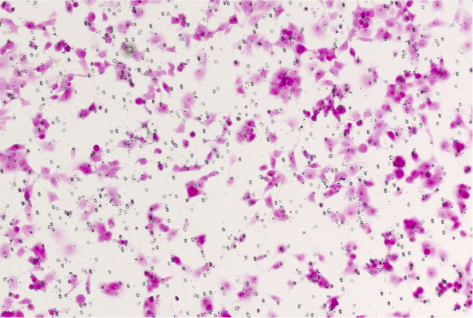

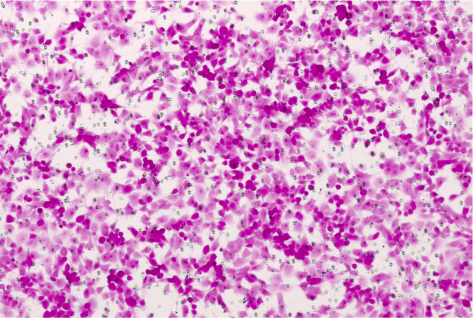


Transwell-Invasion

1st

MIA PaCa-2

PBS rANGPTL4


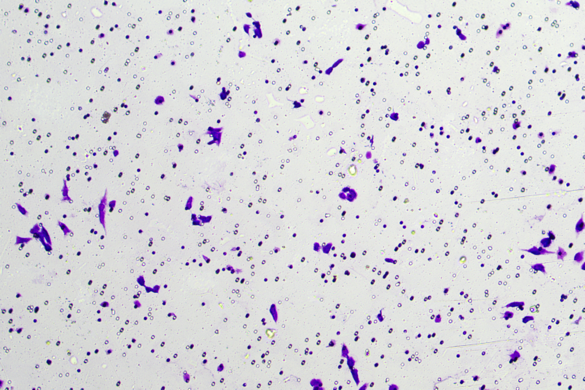

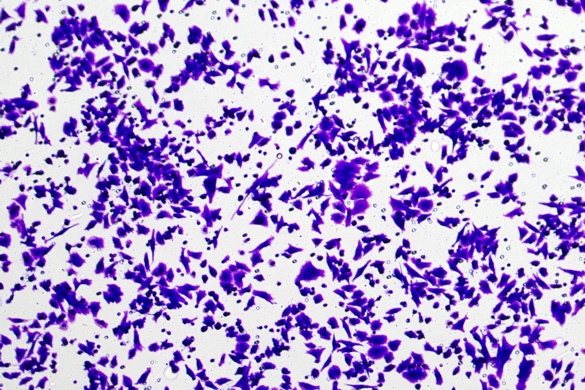


PANC-1

PBS rANGPTL4


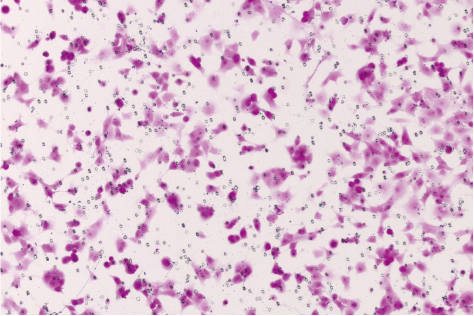

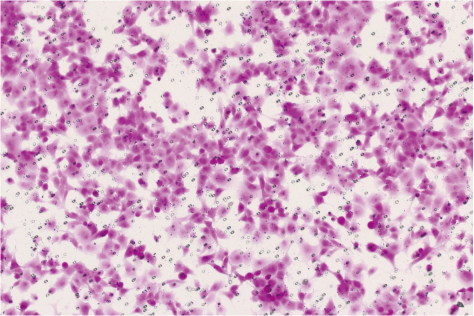


2nd

MIA PaCa-2

PBS rANGPTL4


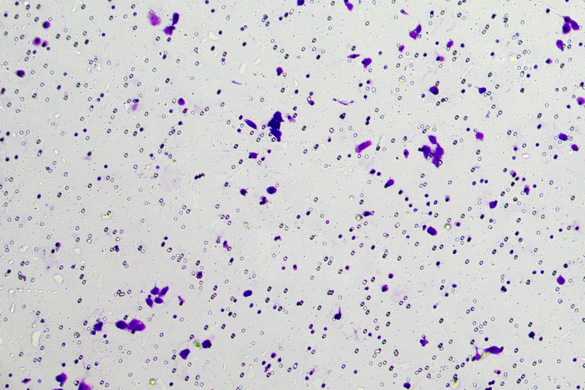

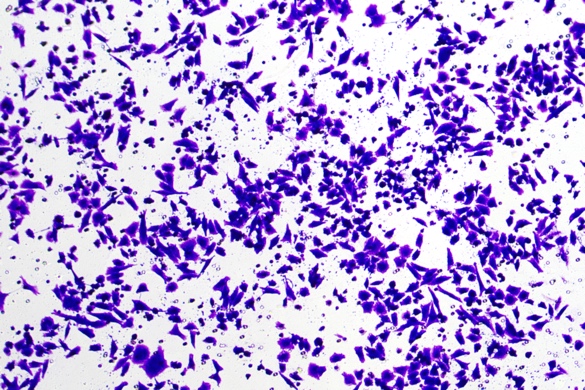


PANC-1

PBS rANGPTL4


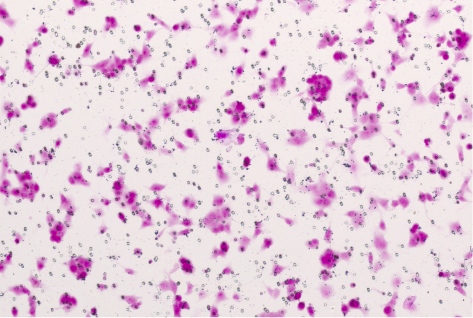

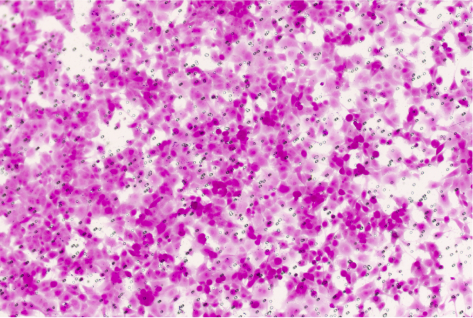


3rd

MIA PaCa-2

PBS rANGPTL4


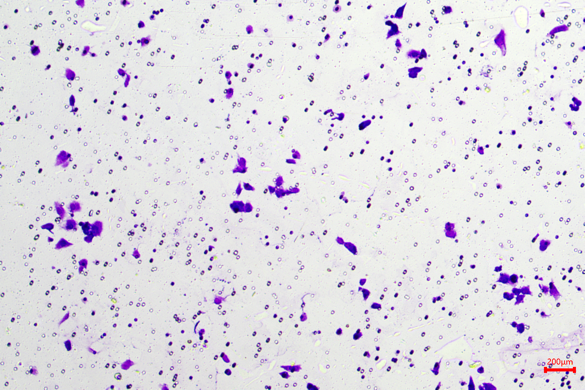

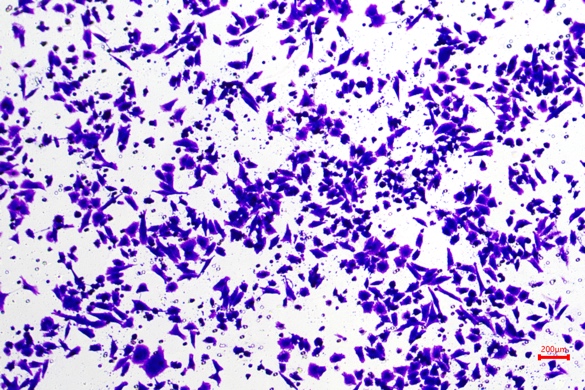


PANC-1

PBS rANGPTL4


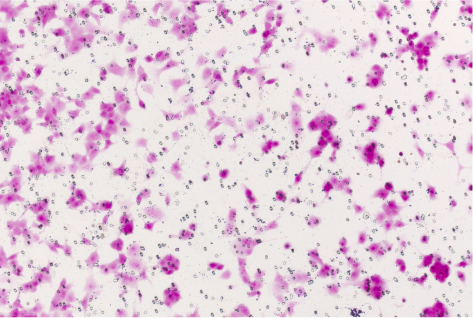
 **
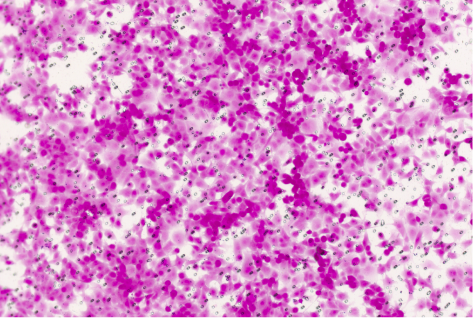
**
