# Supplementary figures and images for: Analysis of molecular subtypes and prognostic signature of senescence-associated secretory phenotype in pancreatic cancer
Source: PeerJ. 2026 Jan 6;14:e20476. doi: 10.7717/peerj.20476 (PMC12786131; doi:10.7717/peerj.20476)

# MIA PaCa-2

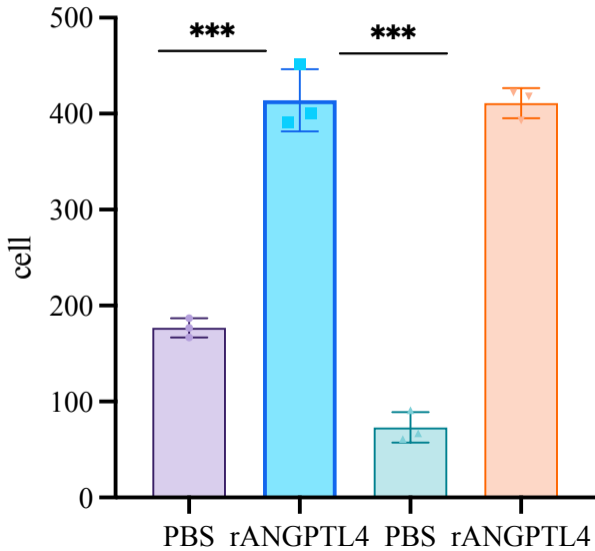

Supplement: Supplemental Information 3 — The raw data in PZFX format was created using GraphPad Prism (Version 10), downloaded from the official website ( https://www.graphpad.com/features). [file peerj-14-20476-s003.zip › Raw data/MIA-Migration.pdf]

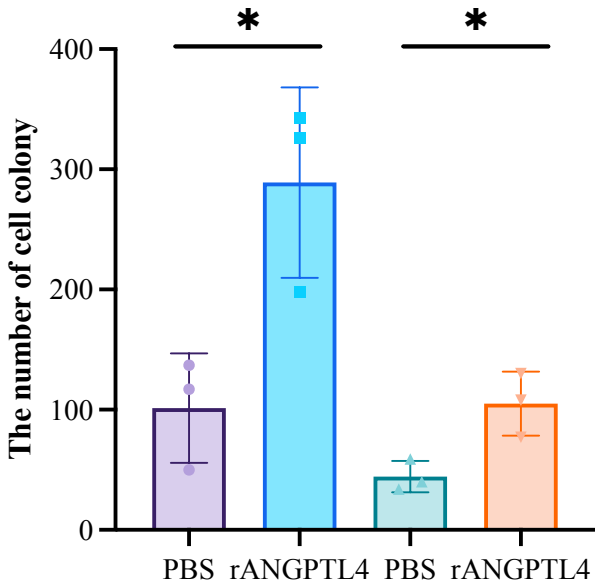

Supplement: Supplemental Information 3 — The raw data in PZFX format was created using GraphPad Prism (Version 10), downloaded from the official website ( https://www.graphpad.com/features). [file peerj-14-20476-s003.zip › Raw data/colony formation.pdf]

# PANC-1

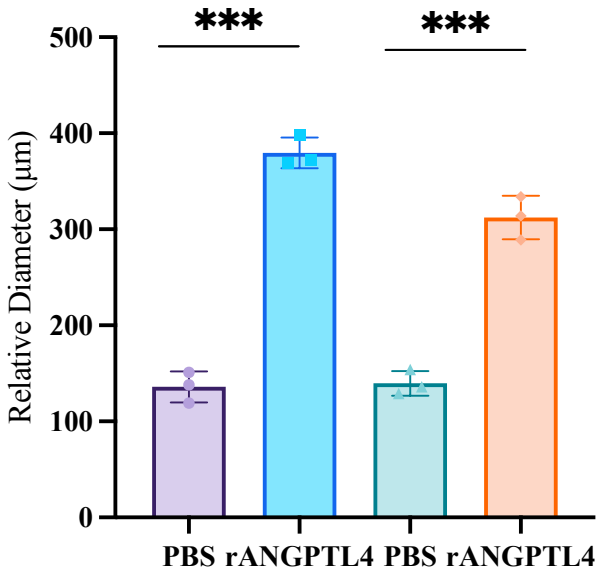

Supplement: Supplemental Information 3 — The raw data in PZFX format was created using GraphPad Prism (Version 10), downloaded from the official website ( https://www.graphpad.com/features). [file peerj-14-20476-s003.zip › Raw data/PANC-Migration.pdf]
